# Optimization of quenched fluorescent peptide substrates of SARS-CoV-2 3CL^pro^ main protease (Mpro) from proteomic identification of P6—P6' active site specificity

**DOI:** 10.1128/jvi.00049-24

**Published:** 2024-05-14

**Authors:** Hugo Cesar Ramos de Jesus, Nestor Solis, Yoan Machado, Isabel Pablos, Peter A. Bell, Reinhild Kappelhoff, Peter M. Grin, Carlos A. Sorgi, Georgina S. Butler, Christopher M. Overall

**Affiliations:** 1 Centre for Blood Research, Life Sciences Centre, University of British Columbia, Vancouver, British Columbia, Canada; 2 Department of Oral Biological and Medical Sciences, University of British Columbia, Vancouver, British Columbia, Canada; 3 Department of Biochemistry and Molecular Biology, The University of British Columbia, Vancouver, British Columbia, Canada; 4 Department of Chemistry, Faculty of Philosophy, Sciences and Letters at Ribeirão Preto, University of São Paulo, São Paulo, Brazil; 5 Yonsei Frontier Lab, Yonsei University, Seoul, Republic of Korea; Loyola University Chicago - Health Sciences Campus, Maywood, Illinois, USA

**Keywords:** COVID-19, SARS-CoV-2, 3CL^pro^ main protease, Mpro, proteomics, PICS, oxidised methionine, P' side, structure activity relationship, peptide assays

## Abstract

SARS-CoV-2 3C-like main protease (3CL^pro^) is essential for protein excision from the viral polyprotein. 3CL^pro^ inhibitor drug development to block SARS-CoV-2 replication focuses on the catalytic non-prime (P) side for specificity and potency, but the importance of the prime (P') side in substrate specificity and for drug development remains underappreciated. We determined the P6–P6' specificity for 3CL^pro^ from >800 cleavage sites that we identified using Proteomic Identification of Cleavage site Specificity (PICS). Cleavage occurred after the canonical P1-Gln and non-canonical P1-His and P1-Met residues. Moreover, P3 showed a preference for Arg/Lys and P3' for His. Essential H-bonds between the N-terminal Ser1 of protomer-B in 3CL^pro^ dimers form with P1-His, but not with P1-Met. Nonetheless, cleavage occurs at P1-Met456 in native MAP4K5. Elevated reactive oxygen species in SARS-CoV-2 infection oxidize methionines. Molecular simulations revealed P1-Met_OX_ forms an H-bond with Ser1 and notably, strong positive cooperativity between P1-Met with P3'-His was revealed, which enhanced peptide-cleavage rates. The highly plastic S3' subsite accommodates P3′-His that displays stabilizing backbone H-bonds with Thr25 lying central in a “‘threonine trio” (Thr24-Thr25-Thr26) in the P'-binding domain I. Molecular docking simulations unveiled structure-activity relationships impacting 3CL^pro^-substrate interactions, and the role of these structural determinants was confirmed by MALDI-TOF-MS cleavage assays of P1'- and P3'-positional scanning peptide libraries carrying a 2nd optimal cut-site as an internal positive control. These data informed the design of two new and highly soluble 3CL^pro^quenched-fluorescent peptide substrates for improved FRET monitoring of 3CL^pro^ activity with 15× improved sensitivity over current assays.

**IMPORTANCE:**

From global proteomics identification of >800 cleavage sites, we characterized the P6–P6' active site specificity of SARS-CoV-2 3CL^pro^ using proteome-derived peptide library screens, molecular modeling simulations, and focussed positional peptide libraries. In P1', we show that alanine and serine are cleaved 3× faster than glycine and the hydrophobic small amino acids Leu, Ile, or Val prevent cleavage of otherwise optimal non-prime sequences. In characterizing non-canonical non-prime P1 specificity, we explored the unusual P1-Met specificity, discovering enhanced cleavage when in the oxidized state (P1-Met_OX_). We unveiled unexpected amino acid cooperativity at P1-Met with P3′-His and noncanonical P1-His with P2-Phe, and the importance of the threonine trio (Thr24-Thr25-Thr26) in the prime side binding domain I in defining prime side binding in SARS-CoV-2 3CL^pro^. From these analyses, we rationally designed quenched-fluorescence natural amino acid peptide substrates with >15× improved sensitivity and high peptide solubility, facilitating handling and application for screening of new antiviral drugs.

## INTRODUCTION

Severe acute respiratory syndrome (SARS)-coronavirus 2 (CoV-2) is the etiologic agent of coronavirus disease 2019 (COVID-19) in the first documented coronavirus pandemic (1). With SARS-CoV-2 now endemic, infected human reservoirs of susceptible under-vaccinated populations, including children and the immunocompromised, and new variants of concern (VOCs) exhibiting immune- and vaccine-escape potential highlight the likelihood of the emergence of further VOCs. The looming health challenge for COVID-19 caused by new VOCs is to improve antiviral drugs for treatments and that can be pre-positioned as scaffolds for rapid drug development in future coronavirus outbreaks.

SARS-CoV-2 is a positive single-stranded RNA virus encoding four structural proteins (spike, envelope, membrane, and nucleocapsid) and 15–16 non-structural proteins (nsps), including two viral cysteine proteases: nsp5 3C-like protease (3CL^pro^), also known by the subjective term, main protease (Mpro), and nsp3 papain-like protease (PL^pro^) (2). The SARS-CoV-2 proteases excise proteins from two frameshift viral polyproteins encoded by ORF1a and ORF1ab. We have used Terminal Amino Isotopic Labeling of Substrates (TAILS) proteomics to discover and validate >150 human host cell substrates of 3CL^pro^ that promote SARS-CoV-2 replication and circumvention of innate immune defenses and antiviral xenophagy, including cleavage-inactivation of galectin-8 to avoid xenophagy destruction (3). 3CL^pro^ was validated early in the pandemic as a therapeutic target — effective treatment of COVID-19 was achieved by inhibiting 3CL^pro^ activity with Paxlovid, a combination drug of Nirmatrelvir and Ritonavir (4), emphasizes the importance of the protease in viral replication and the concern that mutant 3CL^pro^ (5) may evolve reduced susceptibility to Nirmatrelvir. A next-generation high-potency anti-proteolytic antiviral drug, Ibuzatrelvir (PF-07817883), with low off-target effects and not requiring Ritonavir to inhibit cytochrome P450-3A4, is in clinical trials by Pfizer to treat infection and prevent transmission. However, high-throughput screening (HTS) for follow-up inhibitors is hampered by poor solubility and handling properties of even the best current quenched fluorescent (QF) peptide substrates of 3CL^pro^ (6).

Substrate cleavage requires the amino acids flanking the scissile bond on the non-prime (P) side and distal prime (P') side to fit the protease S and S' subsites, respectively. The essential role of interactions between Ser1 of Protomer B in SARS-CoV-2 3CL^pro^ dimers with P1-Gln in the S1 subsite for substrate stabilization and peptide bond scission was first described by Kneller et al. (7). Early in the pandemic, Rut et al. (6) employed a combinatorial library of natural and non-natural amino acids to determine the P side specificity of 3CL^pro^, which showed similarities and differences with the SARS-CoV (−1) and MERS 3CL^pro^. Similarly, active site structural characterization has mainly been directed to the P side to inform the development of antiviral protease inhibitors. In contrast, the contribution of the P' prime side has often been overlooked. Here, we describe the complete P6–P6' active site substrate specificity of SARS-CoV-2 3CL^pro^ that we determined from >800 cleavage sites identified in three different proteome-derived peptide libraries using Proteomic Identification of Cleavage site Specificity (PICS) (7, 8). Employing focused positional peptide libraries and molecular dynamics modeling of the best-fitting sequences, we identified undescribed P'-side natural amino acid residue preferences and their structural determinants in 3CL^pro^ interactions. Crucial P'-side specificity determinants that we identified include a highly plastic P3' subsite moulding the physicochemical envelope of the active site. These data informed the development of new highly soluble, high-efficiency fluorescence resonance energy transfer (FRET) peptide substrates for improved assays of 3CL^pro^ activity, such as for HTS screening. Moreover, peptide cleavage after P1 methionine in a preferred oxidized form (Met_OX_) was established further emphasizing the relevance of oxidative stress in viral infections. These findings should inform med-chem improvement of new small- molecule inhibitor compounds potent against SARS-CoV-2 3CL^pro^.

## MATERIALS AND METHODS

### Expression and purification of SARS-CoV-2 3CL^pro^ active and inactive mutants

We used synthetic DNA encoding SARS-CoV-2 3CL^pro^ (nsp5); YP_009725301.1 (protein ID), NC_45512.2 (whole SARS-CoV-2 genome) cloned into pET-21b (+) (3). A Gln306Ala (Q306A) mutation eliminated the C-terminal 3CL^pro^ autoproteolytic site (Gln306↓Gly), which otherwise removed the C-terminal tag comprising of a Gly_3_ flexible linker, a Factor Xa cut site, Gly_2_ linker, 3× FLAG-tag, Gly_2_ linker, Myc-tag, Gly_2_ linker, and finally a His_6_-tag. Our protein expression vector was deposited to Addgene.org as #177334, pET21b(+)_SARS-CoV-2_3CLpro-Q306A. A catalytic Cys145 to Ala (C145A) mutation was made to generate the control inactive mutant protease. This protein expression vector was deposited to Addgene.org as #177335, pET21b(+)_SARS-CoV-2_3CLpro-C145A-Q306A (3).

The wild-type 3CL^pro^ and inactive mutant proteases were expressed in *Escherichia coli* BL21(DE3)pLysS (Thermo Fisher Scientific) and the recombinant proteins were purified by immobilized metal affinity chromatography as we described in Pablos et al. (3). Anti-FLAG immunoreactive protease-containing fractions were pooled and dialyzed against assay buffer (150 mM NaCl, 2 mM DTT, 1 mM EDTA, 0.05% Brij 35, 50 mM Tris-HCl, pH 6.9), snap-frozen in liquid N_2_, and stored at –80°C.

### Quenched fluorescent peptide cleavage assays

Quantification of 3CL^pro^ activity was by using the SARS-CoV-2 3CL^pro^-specific quenched fluorescent peptide (Ac-Abu-Tle-Leu-Gln-ACC) at 20 µM as described by Rut et al. (6). Fluorescence on cleavage was measured at λex 320 nm and λem 460 nm using a POLARstar optima (BMG LABTECH) microplate reader. The pH optimum for 3CL^pro^ activity was determined over a pH range of 5–10, and the dimerization concentration was determined by kinetic measurements using the quenched fluorescent peptide substrate.

We synthesized new SARS-CoV-1 3CL^pro^ quenched fluorescent peptides for assay with the fluorophore 7-methoxycoumarin-4-acetyl (Mca) and the quencher 2,4-dinitrophenyl (Dnp) (Mca-AVLQ↓SGFR{Lys(Dnp)}RR-NH_2_) measured at λex = 320 nm and λem = 405 nm. From cleavage site specificity analyses, we designed the following sets of synthetic peptide substrates QFS1: Mca-RVALQ↓**X**AHYK(Dnp)RR, where P1′-**X** is Ser (QFS1-S1'), Ala (QFS1-A1'), or Gly (QFS1-G1'); and the optimized quenched fluorescent QFS2-peptide substrates , Mca-VRLQ↓SK(Dnp)RR (QFS2-S1'), and Mca-VRLQ↓AK(Dnp)RR (QFS2-A1') (GenScript).

### Proteome library preparation

*E. coli* strain K12 lysates were used to prepare proteome-derived peptide libraries as described (8, 9). As *E. coli* does not extensively modify its proteins, it provides suitable proteomes to determine protease specificity. Protein supernatants were reduced with 10 mM dithiothreitol (DTT) for 60 min at 37°C and alkylated with 20 mM iodoacetamide for 30 min at room temperature in the dark. Lysates were quenched with 20 mM DTT and precipitated by mixing 5 mL of lysate with 30 mL of ice-cold acetone:methanol (8:1) and incubating at –80°C for 16 h. Proteome pellets were collected by centrifugation at 9,000 × x*g* for 15 min at 4°C, washed with ice-cold acetone, dissolved in 6 M GuCl, and quantified by A_280_ nm.

To prepare the trypsin library, 10 mg of protein was diluted 10-fold in 200 mM HEPES, pH 8.0. For the GluC library, 10 mg of protein was diluted 10-fold in 200 mM phosphate-buffered saline, pH 7.5. For the lysargiNase (10) library, 10 mg of protein was diluted 10-fold in 200 mM HEPES, pH 8.0, and CaCl_2_ was added to 10 mM. For each library, sequencing-grade enzyme was added: 100 µg trypsin (Thermo Fisher Scientific), 200 µg GluC (Thermo Fisher Scientific), or 200 µg lysargiNase (https://www.ibmb.csic.es/en/department-of-structural-and-molecular-biology/proteolysis-lab/lysarginase/) and incubated at 37°C for 16 h with rotation. The digested protein samples were heated at 80°C for 10 min to deactivate proteases, followed by incubation with 10 mM EDTA and 1 mM 4-(2-aminoethyl)benzenesulfonyl fluoride hydrochloride. Peptide mixtures were adjusted to pH 8.0, and then free amines dimethylated with 100 mM formaldehyde and 80 mM sodium cyanoborohydride at 37°C for 16 h. Dimethylated peptides were quenched with 150 mM Tris-HCl, pH 8.0, for 45 min at 37°C and then acidified to pH 3 using formic acid with excess gas allowed to bubble off for 30 min. Peptide samples were purified by solid-phase extraction using C18 SPE cartridges (Waters). Cartridges were activated with 100% acetonitrile (MeCN), equilibrated with 0.1% formic acid, followed by sample loading. Flowthrough peptides were collected and reloaded. Cartridges were washed thrice with 0.1% formic acid (6 volumes). Dimethylated peptides were eluted twice with 70% MeCN, 0.1% formic acid into Protein LoBind tubes (Eppendorf) and lyophilized to complete dryness. Dimethylated peptide libraries were resuspended in water, quantified by *A*_280_ nm, aliquoted, and frozen at –80°C until PICS assays.

### Proteomic identification of cleavage substrates

In PICS, 3CL^pro^-cleaved neo-N-termini display a free N-terminal amine that is reactive with NHS-biotin, which is used to purify the prime side cleavage products. To do so, the N-terminal-blocked proteome-derived peptide libraries were prepared for assay as follows: 300 µg of each library was resuspended in 3CL^pro^ assay buffer [50 mM Tris, 150 mM NaCl, 2 mM DTT, 1 mM EDTA, 0.05% Brij-35, at pH 6.9, which we determined was the pH optimum for the protease (Fig. S1)] in Protein LoBind tubes at 2 mg/mL. 3CL^pro^ or inactive 3CL^pro^-Cys145Ala as control were added to each library at the dimerization concentration that we determined to be 0.8 µM, gently mixed, and incubated at 37°C for 16 h. Digestions were stopped by heat inactivation. Reduced cleavage of dimethylated lysine-containing peptides by proteases cleaving with Lys specificity is a constraint of PICS (9).

A stock of 10 mM sulfo-NHS-biotin was prepared in DMSO, added to each digested sample to a final concentration of 0.5 mM, and incubated with rotation at 25°C for 2 h. High-capacity streptavidin-Sepharose (1.5× reaction volume) was equilibrated 5 times in 50 mM HEPES, 150 mM NaCl, pH 7.5. The biotinylated products were mixed with washed slurry and vortexed for 30 min at 25°C, after which the mixture was transferred to a spin column (~500 µL capacity) with a filter of ~10 µm pore size. Spin filters were centrifuged at 100 × *g* for 15 s or until all contents passed through without drying the slurry. Flowthroughs were reapplied and centrifuged again. The resin was washed 10 times and centrifuged with 500 µL of 50 mM HEPES, 150 mM NaCl, pH 7.5. Spin filters were plugged, and elution buffer (50 mM HEPES, 20 mM DTT, pH 7.5) was added to the slurry and mixed with agitation for 1 h at 25°C. Eluates were collected in fresh tubes by removing the column plug and centrifugation at 150 × *g* for 15 s or until contents had passed through without drying the column. A second elution was performed using fresh elution buffer; eluates were combined and then acidified to pH 3 with formic acid. Peptides were desalted and concentrated using C18 SPE cartridges as described above. Peptides were lyophilized to complete dryness and analyzed by RP-LC-MS/MS.

### RP-LC-MS/MS

Purified peptides were resuspended in 5 µL buffer A (0.1% formic acid, 99.9% H_2_O) for injection onto an Easy nLC-1000 (Thermo Scientific) connected inline to a Bruker Impact II Q-TOF mass spectrometer (Bruker Daltonics). Peptides were loaded onto an in-house-packed analytical column (25 cm × 75 µm ID, 1.9 µm C18AQ ReproSil-Pur, Dr. Maisch) at 800 bar using buffer A heated by a column oven at 50°C and then eluted with a linear gradient of buffer B (0.1% formic acid, 99.9% MeCN) over 90 min at 200 nL/min from 2% buffer B to 24% buffer B, then washed with 95% buffer B for 5 min before re-equilibration with buffer A. Eluted peptides were ionized by electrospray and analyzed in positive ion mode, source voltage 1.5 kV and 150°C, with nanoBooster using MeCN at 3.0 L/min and then analyzed by data-dependent acquisition. Ions were scanned between 150–1,750 *m*/*z* at 5 Hz, and then the top 12 most intense precursor peptide ions (charges 2–5) were selected for fragmentation by collision-induced dissociation, and the product ions were scanned at 8 Hz for counts above 1.2 × 10^3^ and at 12 Hz for counts above 2.5 × 10^5^ and actively excluded for MS/MS after 1 spectrum for 30 s but reconsidered if the intensity was 5-fold higher than the previous intensity. Spectra were converted into .mgf files for database searching.

### Database searching and data analysis

Byonic v3.8.1 (Protein Metrics, San Carlos, CA USA; version PMI-Byonic-Com:v3.8.1) was used to search mgf files. Parameters for searching were: MS1 tolerance 15 ppm, MS2 tolerance 0.04 Da, protein database *E. coli* K12 (UniProt-proteome UP000000625) with a 1% false discovery rate using a concatenated reverse database including common contaminants. Static modifications: carbamidomethylation of cysteine (+57.021464 @C), dimethylation of lysine (+28.031303 @K). Variable modifications: oxidation of methionine (+15.994915 @M), dimethylation of N-termini (+28.031300 @NTerm), thioacylation of N-termini (+87.998285 @NTerm), deamidation of asparagine (+0.984016 @N). Sulfone was not searched for as a variable modification. Depending on the enzyme library used, the cleavage specificity was defined: for trypsin (semi-cleavage C-terminal to Arg and Lys), for GluC (semi-cleavage C-terminal to Asp and Glu), for lysargiNase (semi-cleavage N-terminal to Arg and Lys).

Peptides that were thioacylated and semi-cleaved (i.e., biotinylated peptides resulting from 3CL^pro^ cleavage) were considered for sequence analysis by WebPICS (https://webpics.clip.msl.ubc.ca) (11). The amino acid occurrences from P6–P6' were normalized for natural abundance in the *E. coli* K12 reference proteome, and their occurrence at each position (P6–P6') was calculated (*p*-value ≥ 0.05). Amino acids with a >2-fold occurrence over the normalizsed baseline were plotted as heat maps using GraphPad Prism version 9.0.0.121 (GraphPad Software Inc., San Diego, CA). WebPICS outputted the P4–P4' sequences for cut site motif analysis using IceLogo version 1.3.8. Amino acid synergism/cooperativity analysis was performed by using fixed amino acid subsite position analyses in WebPICS to establish any positive or negative amino acid cooperativity >10% frequency versus independence between the fixed amino acid and amino acids in other positions.

### 3CL^pro^ cut-site kinetics by MALDI-TOF/TOF-MS

Peptides with the following sequences: VALQGAHRVALQ**X**AHYR (**X** = Leu, Val, or Ile), RASVALQGA**X**YSAR (X = His, Thr, or Ala), RASVALQ**X**AHYSAR (**X** = Ser, Ala, or Gly), and AAVALQ**X**AHHYAYR (**X** is one of each 20 natural amino acids) were synthesized (GenScript). Peptides (50 µM) were diluted in 3CL^pro^ assay buffer, pH 6.9, and incubated with 3CL^pro^ (1:50 molar ratio, E:S) in a 25 µL final volume at 37°C in a humidified chamber for 5, 15, 30, 60, 120, 240 min. At each time point, 0.5 µL of the assay reactants was spotted on a matrix-assisted laser desorption/ionization (MALDI) plate pre-spotted with alpha-cyano-4-hydroxy-cinnamic acid matrix solution (10 mg/mL in 50/50/0.1% water/acetonitrile/formic acid) after which 0.5 µL matrix solution was immediately added. The spotted samples were desalted in an ice-cold 0.1% formic acid bath by immersing the plate, which was then loaded into a MALDI-TOF/TOF 4700 Proteomics Analyzer (Applied Biosystems) equipped with a 335 nm Nd:YAG laser (200 Hz) operating in positive ion mode. MALDI spectra were analyzed using Applied Biosystem Data Explorer, version 4.5. The apparent (app) ^app^(*k*_cat_/*K*_M_) was estimated as before (3) under the assumption of a first-order reaction where


$$t\frac{\text{1}}{\text{2}}=\frac{ln2}{k_{\text{cat}}/K_{\text{M}}\left[E_{\text{0}}\right]}$$

### P1 methionine oxidation and MS/MS analysis

The peptide AAVALM↓SAHHYAYR was synthesized and the methionine was oxidized by incubating 5 µL of 10 mM peptide (0.05 µM) with 5 µL 30% aqueous H_2_O_2_ (25 µM, 500× excess) in 50 µL glacial acetic acid for 15 min at 22°C. 100% conversion of Met to Met_OX_ was confirmed by mass spectrometry as follows. The peptide sample was desalted on an Oasis HLB cartridge and eluted with 1 mL MeCN, 0.1% formic acid, and 50 µL was then diluted in 150 µL H_2_O and measured by direct infusion on an Impact II Q-TOF mass spectrometer (Bruker-Daltonics) in positive ion mode. For cleavage assays, the acetonitrile was removed from the oxidized peptide by SpeedVac and solubilized in 50 mM Tris-HCl, 1 mM EDTA, 50 mM NaCl, pH 6.9 in the absence of DTT. To 200 µg/mL P1-Met-peptide or P1-Met_OX_-peptide, 80 µM 3CL^pro^ or 3CL^pro^ (Cys145Ala) as control were added to a final 1:100 (E:S ratio), incubated and analyzed by MALDI-TOF-MS as described above.

### Molecular docking simulations

Peptide-3CL^pro^ molecular docking simulations were performed using Rosetta FlexPepDock *ab-initio* protocol (12) in the Rosetta software suite (13). The 3CL^pro^ structure (PDB: 6XHU) (14) was prepared for docking calculations by running the Rosetta relax application using flags listed in Supporting Information. The starting backbone conformation of an intact peptide spanning a canonical P4–P4′ that we identified by PICS (VALQ↓GAHY) was created as a preliminary extended structure truncated at both N- and C-termini using the BuildPeptide Rosetta application. A fragment library of trimer and pentamer backbones was generated from known PDB structures based on sequence similarity and predicted secondary structure. FlexPepDock *ab initio* simulations were performed of the 3CL^pro^ active site with the extended peptide structure positioned 15 Å away. Fast, low-resolution modeling was performed 50,000 times with the side chains represented as single-centroid spheres. High-resolution analysis was then performed using a full-atom energy function, which enables full flexibility for all peptide and receptor side chains (15). A flat harmonic function (Supporting Information) penalized models where the Euclidean distance between the P1-Cα and Ser1-Sγ was >4 Å. The Rosetta Interface score (I_sc) (16) was calculated by subtracting the energy of 3CL^pro^ and the peptide in isolation from the total energy of the complex. The 500 lowest-scoring models based on Rosetta total energy were selected, within which the model with the highest structural similarity was selected as the representative model. Other parent peptides of cleavage products identified by PICS that we modeled were SRLH↓SYSS, SKLM↓SENT, and SKLM_OX_↓SENT versus ARLQ↓AMAP, AALQ↓AVNS, VVVQ↓AASG, AILQ↓NATS and SDLQ↓STQA.

## RESULTS

### Incubation of PICS libraries with 3CL^pro^ yields cleaved peptides identifiable by LC-MS/MS

**Fig 1:**
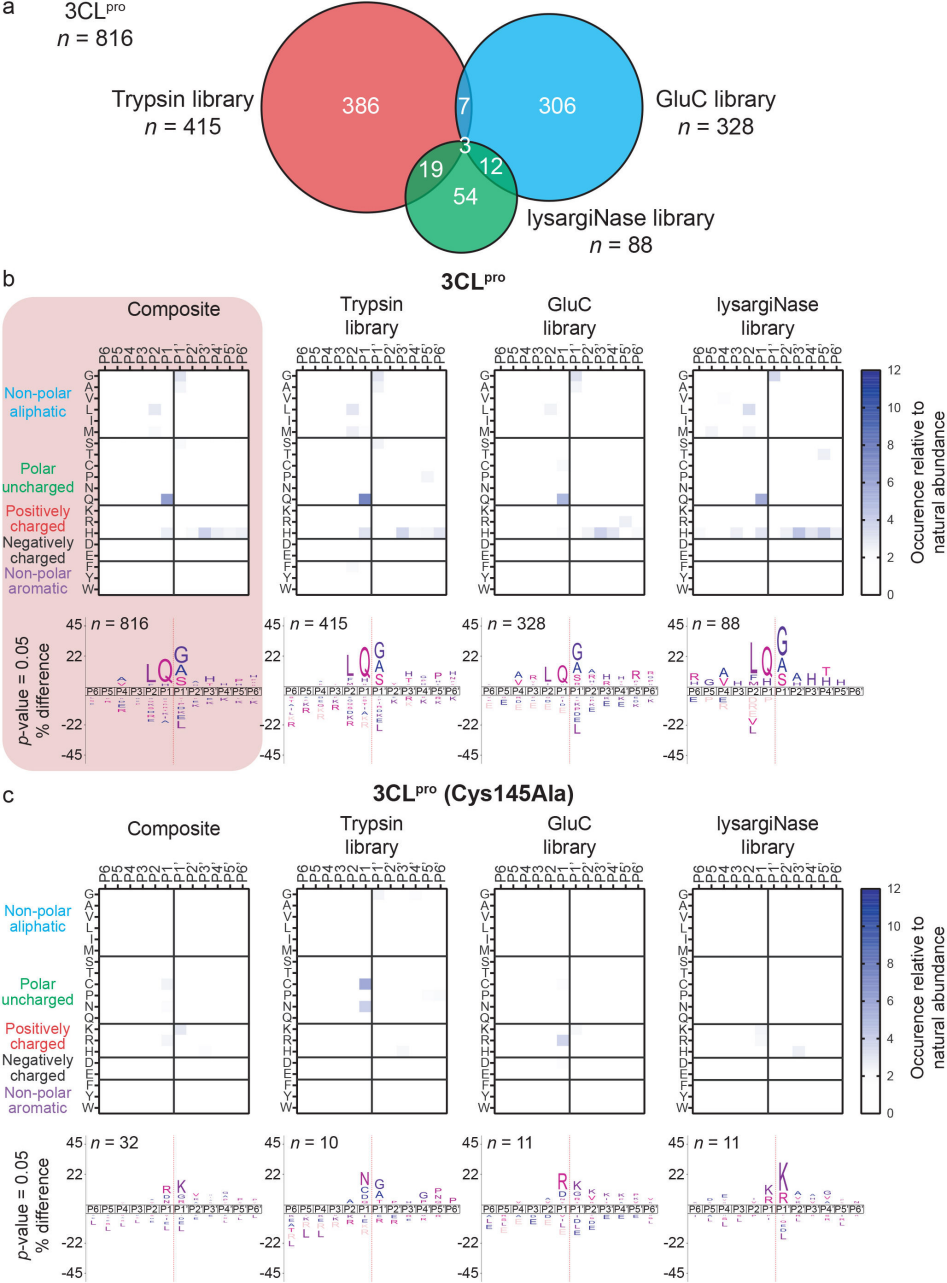
Protease specificity of SARS-CoV-2 3CL^pro^ determined by PICS assays. (a) Venn diagram showing the distribution of the 816 cleavage sites identified after incubating active SARS-CoV-2 3CL^pro^ with proteome-derived peptide libraries generated by one of the three proteases shown (trypsin, GluC, LysargiNase) (*N* = 2). Heatmaps with their associated Icelogos for each PICS library and the composite data compiled from the three libraries for (b) active 3CL^pro^ and (c) control catalytic inactive mutant 3CL^pro^ (Cys145Ala) experiments. Heatmaps show sites with an occurrence value >2-fold. Highlighted in red is the composite motif across all libraries that provides the substrate sequence specificity for 3CL^pro^ determined from *n* = 816 cleavage sites identified by PICS. See also Fig. S2 for matching data for each of the two independent replicates of the assays.

Digestion of proteomes with proteases of differing sequence specificities (trypsin, GluC, and lysargiNase) (10) yielded three separate proteome-derived peptide libraries, each distinguished by characteristic amino acids at the C-terminus (Arg/Lys for trypsin, Glu/Asp for GluC) or N-terminus (Arg/Lys for lysargiNase). After dimethylation to block primary amines, the libraries were assayed for susceptibility to cleavage by recombinant SARS-CoV-2 3CL^pro^. Cleaved neo-N-terminal products displaying a free α-amine at the N-terminus were biotinylated to detect 3CL^pro^-cleaved neo-N-terminal peptides after streptavidin enrichment. From the identity of the biotinylated peptides, which were the P' sequences of the cleavage sites, 816 cleavage sites spanning P6–P6' were identified across the three libraries in *N* = 2 separate independent experiments (Fig. 1; Fig. S2). Using three digestion enzymes to generate the PICS proteome libraries greatly expanded the number of cleavage sites identified (Fig. 1a): Digestion of the trypsin, GluC, and lysargiNase libraries with 3CL^pro^ resulted in 415, 328, and 88 cleavage sites, respectively, with only 41 common sites.

From the occurrence of amino acids at each position, heatmaps were generated (Fig. 1b). The values for the occurrences of all amino acids at all positions were combined by a weighted average into the final composite motif, highlighted in Fig. 1b. Assay robustness was confirmed by all IceLogos showing good agreement across individual replicates and libraries (Fig. S2). As expected, only minor library peptide carryover (*n* = 32) lacking any defined specificity was found in the catalytically inactive 3CL^pro^ (Cys145Ala) samples (Fig. 1c). Since we used the same expression and purification strategies for 3CL^pro^ and 3CL^pro^ (Cys145Ala), which included the use of a cocktail of protease inhibitors, the control data confirm that no co-eluting contaminant proteases were present.

### SARS-CoV-2 3CL^pro^ domain interactions in prime-side substrate recognition

In agreement with previously mapped 3CL^pro^ cleavage motifs in the viral polyprotein (2) and native human cell proteins determined by terminal amine isotopic labeling of substrates (TAILS) (3), the cleavage specificity for 3CL^pro^ is dominated by preferred amino acid residues at three sites, P2, P1, and P1' (6, 17). Unsurprisingly, the non-prime side showed a strong preference for leucine at P2 and glutamine at P1 and for glycine, alanine, and serine at P1'. However, the identification of >800 cut sites allowed for other specificity preferences between P4 and P4' and amino acid cooperativity between subsites in the peptide context to be resolved with statistical significance.

**Fig 2:**
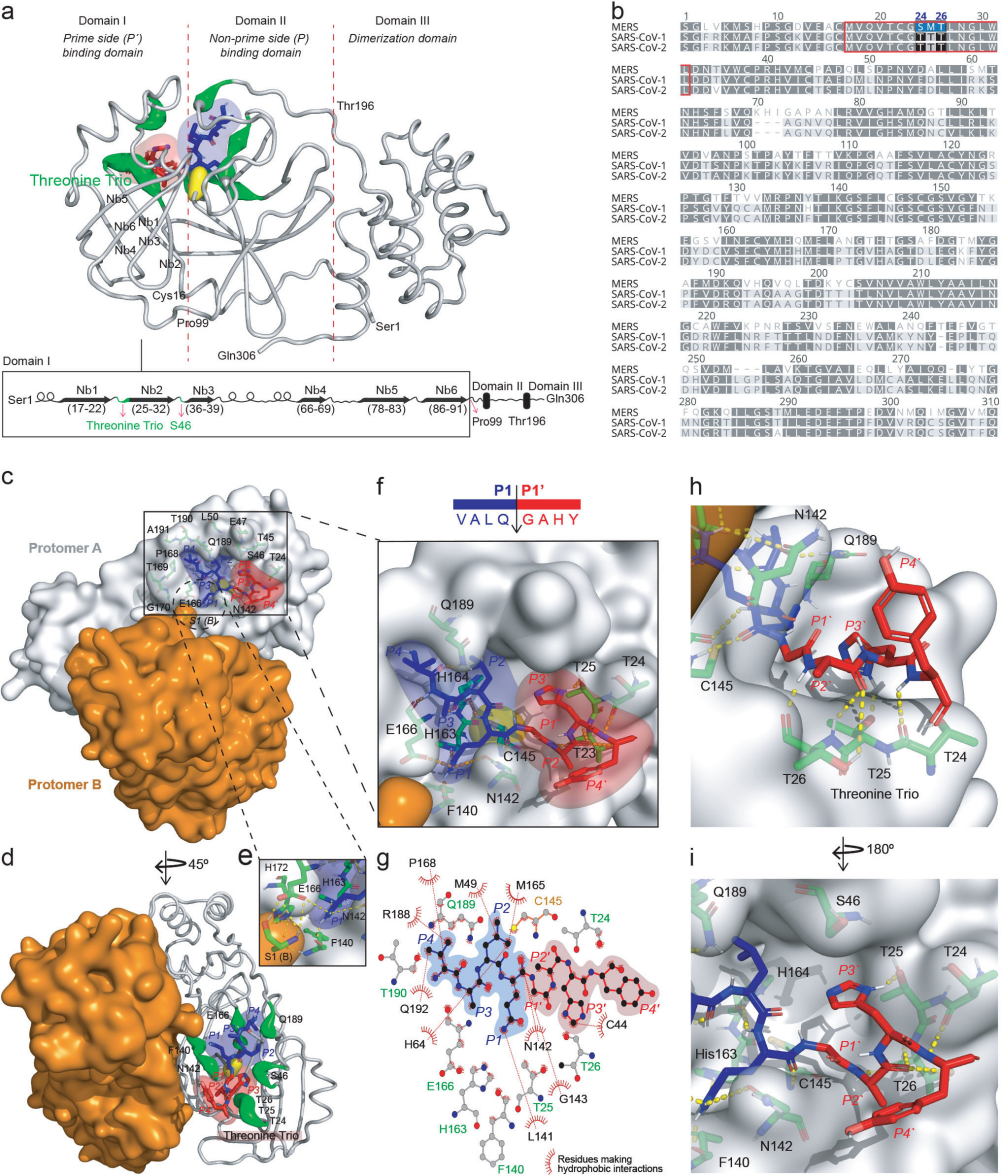
Structure of the top-ranked model of the peptide substrate VALQ↓GAHY docked onto the active site of 3CL^pro^ protomer A (PDB 6XHM). (a) Green putty cartoon representation of 3CL^pro^ protomer A (ribbon representation) residues that interact with the substrate by hydrogen bonding. The substrate interacting amino acid residues and the catalytic cysteine are shown as green and yellow lobes, respectively. The substrate prime side (**P'**) (red sticks) interacts with the 3CL^pro^ catalytic domain I (Ser10–Pro99), whereas the non-prime (**P**) side of the substrate (blue sticks) interacts with domain II (Lys100–Gly182). The C-terminal four residues of dimerization domain III (Asn–Gln 310) are not resolved. A 1D structural representation of Domain I shows the structural features, bounding residues, and location of the P’ substrate interacting threonine trio (Thr24, Thr25, Thr26). (b) Sequence alignment of the 3CL^pro^ from MERS, SARS-CoV-1, and SARS-CoV-2 generated by Clustal Omega showing conservation and divergence of the P' interacting residues in Domain 1 bounded in red and highlighting the threonine trio in SARS CoV-1 and SARS-CoV-2 3CL^pro^, which is absent in the MERS protease (highlighted in blue). (c–i) 3CL^pro^ dimer. Protomers A and B are shown as gray and orange surfaces, respectively. Substrate non-prime (**P**) and prime (**P'**) sides are shown as blue and red sticks, respectively, with the major interacting residues numbered. Green sticks represent interacting 3CL^pro^ amino acid residues. H-bonds are shown as yellow dashed sticks. (d) Green putty cartoon representation of 3CL^pro^ Protomer A residues in Domain I that interact with the substrate by making H-bond interactions. Domains II and III are shown as an orange surface. (e) Enlarged view of the network of intermolecular interactions between 3CL^pro^ Protomer A, Protomer B (Ser1), and the substrate P1-Gln. (f) Enlarged view of the protomer A interactions between 3CL^pro^ active site residues and P4–P4' peptide residues. (g) Hydrophobic interactions between 3CL^pro^ residues and the peptide substrate P4–P4' amino acid residues. (**h, **i) Stabilization of P' amino acid residues by the threonine trio (Thr24-Thr25-Thr26).

To compare the structure-activity relationships for cleaved peptides displaying the preferred amino acids in peptides revealed by PICS and not described for the SARS-CoV-2 3CL^pro^, we constructed nine models of exemplary peptides binding to one of the two 3CL^pro^ protomers in the dimer (PDB 6XHM) through molecular docking simulations (Fig. 2). The I_sc of the final models demonstrated that it is more favorable for 3CL^pro^ and these peptides to interact than to remain separate, e.g., VALQ↓**G**A**H**Y, with a preferred P3′-His, had the lowest (strongest) I_sc = –38.17. The following substrate peptides were modeled with a non-canonical P1-His, SRL**H**↓SYSS and P1-Met, SKL**M**↓SENT and in the oxidized form, Met_OX_, versus the canonical P1-Gln. In addition to the above peptides, P3-Arg was modeled using A**R**LQ↓**A**MAP with a P1′-Ala for comparison with the other well-described P1' amino acid residue by peptides SDLQ↓**S**TQA and AILQ↓**N**ATS. Finally, we compared the preferred P2-Leu (AA**L**Q↓AVNS) with a P2-Val in the cleaved peptide VV**V**Q↓AASG.

Stabilization of the substrate prime side is entirely by domain I of the 3CL^pro^ catalytic domain (residues 10–99), whereas the non-prime side interactions occur through domain II (residues 100–196) (17–19) (Fig. 2a). Three consecutive threonine residues, Thr24, Thr25, and Thr26, which we termed the “threonine trio,” in catalytic domain I are the primary P' stabilizing residues. The P1'–P4' residues extend over the threonine trio, maximizing main-chain interactions between the substrate and 3CL^pro^ (Fig. 2f through i). The main-chain oxygens of Thr24 and Thr26 accept a hydrogen bond from the substrate main-chain nitrogen atom of P4′-Tyr and P2′-Ala at an Euclidian distance of 2.5 and 2.0 Å, respectively. In contrast, the P2′-Ala oxygen forms a bifurcated hydrogen bond with the Thr26 through its main-chain nitrogen (2.8 Å) and side-chain hydroxyl group (3.5 Å) (Fig. 2h and i).

The docking model for VALQ↓GAHY agrees well with experimental evidence from crystallography studies (17–19). Zhao et al. solved the structure of 3CL^pro^ mutant (His41Ala) in complex with six native substrates from SARS-CoV-2 replicase polyproteins and found that the overall structure is almost identical in all six complex structures, with root mean square deviation (RMSD) values ranging from 0.12 Å to 0.32 Å for Cα atoms (17). We aligned the docked PICS peptide with each of the six individual crystal structures (Fig. S3) and observed a substantial-high structural similarity evidenced by the low RMSD for their respective Cα atoms, ranging from 0.16 Å to 0.37 Å (Table S1), validating our approach.

### P1' amino acid preferences

As well-known from the 17 viral polyprotein cut sites of 3CL^pro^ (2), P1' is the key determinant position on the P' side of the cleavage site, with the S1' subsite only accommodating small amino acid residues such as Gly, Ala, and Ser and is prohibitive for charged amino acid residues (Fig. 1b). Our P1' PICS peptide data for SARS-CoV-2 3CL^pro^ are consistent with the cleavage sites in the polyprotein (2) and the reported preferences for the SARS-CoV (−1) protease (20). To quantify minor specificity differences at P1', we synthesized a peptide library by substituting every natural amino acid at P1′-**X** in the sequence AAVALQ↓**X**AHHYAYR. By MALDI-TOF-MS, the ^app^(*k*_cat_/*K*_M_) for 3CL^pro^ cleavage of each substituted peptide confirmed that Ala (1,522.0 ± 7.6 M^−1^s^−1^) and Ser (1,519.1 ± 6.2 M^−1^s^−1^) were equally preferred over Gly (1,079.1 ± 12.6 M^−1^s^−1^) and revealed four minor P1' specificities: His, Phe, Met, and Asn that were cleaved with specificity constants between 45–70 M^−1^s^−1^ (Table 1; Fig. S4).

**TABLE 1:** P1' amino acid preferences of 3CL^pro^ determined by positional scanning of all 20 natural amino acids (X) in the peptide (AAVALQ↓XAHHYAYR)

P1′ amino acid residue	^app^(*k*_cat_*/K*_M_) (M^−1^s^−1^)*^a^*
A	1,522.0 ± 7.6
C	No cleavage
D	No cleavage
E	No cleavage
F	50.3. ± 1.5
G	1,079.1 ± 12.6
H	45.0 ± 0.6
I	No cleavage
K	No cleavage
L	No cleavage
M	65.8 ± 1.4
N	71.8 ± 3.2
P	No cleavage
Q	No cleavage
R	No cleavage
S	1,519.1 ± 6.2
T	No cleavage
V	No cleavage
W	No cleavage
Y	No cleavage

^*a*^ The specificity constants for SARS-CoV-2 3CL^pro^ cleavage of 20 peptides (AAVALQ↓**X**AHHYAYR), where **X** is one of each 20 natural amino acids substituted at P1' to form a library of 20 peptides; ↓, scissile bond. (See also **Fig. S4.**)

**Fig 3:**
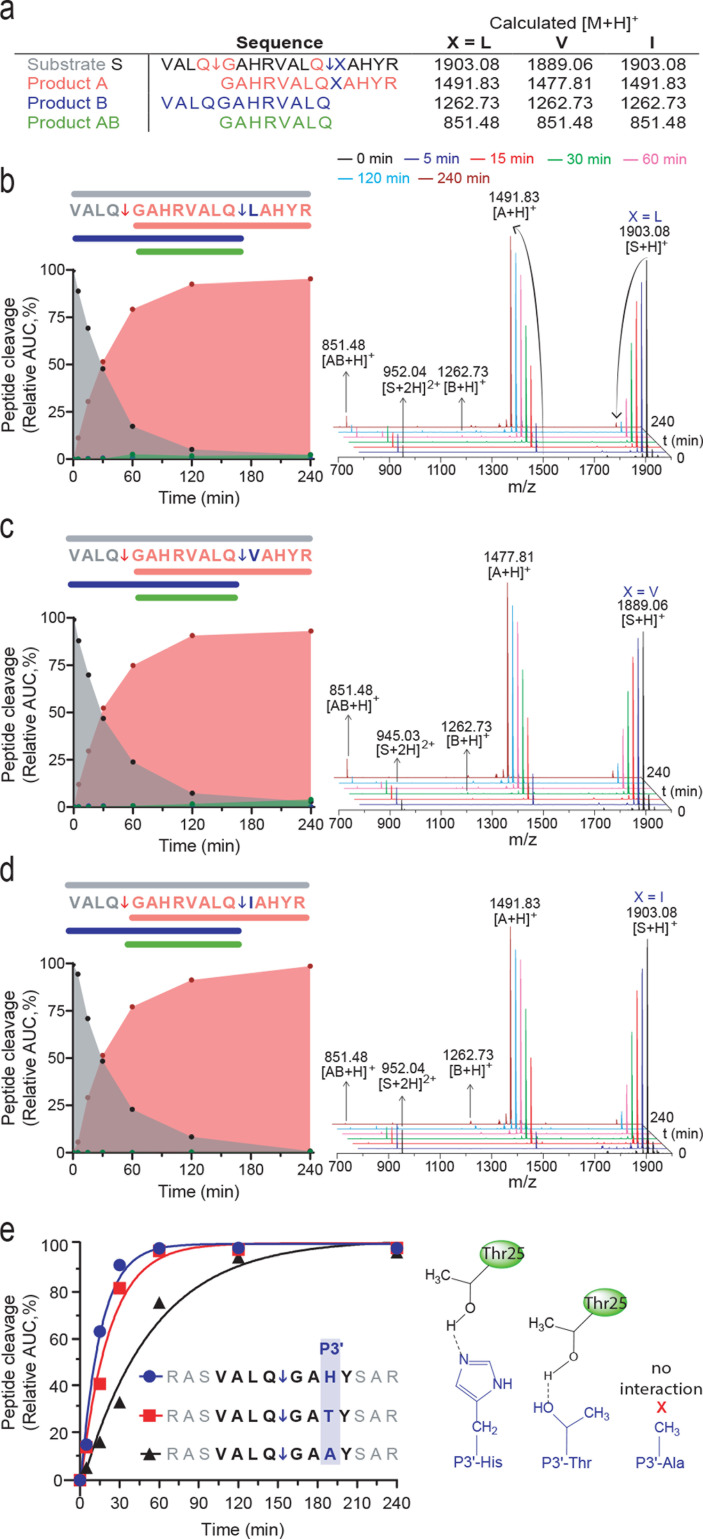
Influence of non-canonical P1' amino acid residues on the cleavage kinetics of 3CL^pro^ with an optimal P sequence. (a) A preferred 3CL^pro^ cleavage site derived from PICS analyses (VALQ↓GAHR) was incorporated as an internal positive control for each peptide containing a non-canonical P1' residue in the otherwise optimal P' cleavage sequence (VALQ↓XAHR) lying C-terminal to the positive control cut site. X was either Leu, Val, or Ile. The calculated [M + H]^+^ of the intact tested peptide and each predicted cleavage product are tabulated. (**b, c and **d) Left, progress curves of peptide cleavage after incubation with 3CL^pro^ (1:50 mole ratio, **E:S**). Right, MALDI-TOF-MS spectra and the detected charge states of the intact and cleaved peptides were measured at times indicated. (e) Influence of hydrogen bond formation between P3' (His, Thr, Ala) and Thr25 on quenched-fluorescent peptide cleavage profiles and kinetic parameters. 3CL^pro^ cleavage sites are indicated by ↓.

From the structural analysis of the substrate peptide VALQ↓GAHY complex with 3CL^pro^ in Fig. 2, P1' selectivity stems from steric hindrance imposed by Thr25, Leu27, and His41 side chains. To confirm the negative effect on 3CL^pro^ activity of having a bulky residue at P1', we synthesized three uniquely designed 17-mer peptides, each displaying two optimal non-prime sequences (underlined) with **X** in the second site substituted for Leu, Val, or Ile: VALQ↓GAHRVALQ↓**X**AHYR. The first site was a preferred cleavage sequence and lay N-terminal to the distal test sequence in the same peptide. This design enabled the normalization of cleavage events within each peptide and between the three members of the focussed library. The second site compared the effect of non-preferred bulky residues at P1'. By quantifying cleavage using MALDI-TOF-MS (Fig. 3a through d) where the P1' **X** was Leu, Val, or Ile, there was minimal cleavage and generation of the cleavage products B = 1,262.73 *m*/*z* or AB = 851.48 *m*/*z*. In contrast, we observed efficient cleavage at the positive control site within the same peptide, where P1' was Gly, generating product A containing the uncut test site with the nonpreferred P1' Val (product A = 1,477.81 *m*/*z*) or Leu/Ile (product A = 1,491.83 *m*/*z*). These data consolidate the evidence found through PICS and molecular modeling that even where the non-prime sequence is optimal, 3CL^pro^ will barely cleave a site with a bulky residue at P1'.

### P3' histidine preference

We found that 3CL^pro^ prefers histidine at P3', which was previously noted for SARS-CoV (−1) 3CL^pro 20^. As revealed by molecular modeling, the P3′-His side chain forms a stabilizing 2.0 Å hydrogen bond with the Thr25 Oγ hydroxyl group (Fig. 2f and i). The S3' subsite is large and plastic enough to accommodate a diverse range of amino acid residues, including bulky residues, with the hydrogen bond formed through Thr25 potentially being the primary P3' stabilizing interaction. We observed great flexibility of the S3’ subsite, with some residues at P1' and P2' swinging into S3' to adopt a lower energy conformational state for the transient complex between the substrate and 3CL^pro^. As discussed later, when the P3' is histidine, 27.5% of these sequences are accompanied by a P1-Met, revealing substrate amino acid cooperativity at a distance. Using a third set of synthetic peptides spanning P4–P4' VALQ↓GA**X**Y, where **X** = His, Thr, or Ala, we observed a direct relationship between the peptide cleavage rate by 3CL^pro^ and the ability of P3' to form a hydrogen bond (Fig. 3e). P3′-Thr was cleaved less efficiently than P3′-His due to the greater propensity of the His side chain to act as a hydrogen bond donor than the Thr hydroxyl group. P3′-Ala displayed the slowest cleavage rate since the non-polar side chain cannot establish hydrogen bonds.

### Structural features of non-prime-side substrate recognition

Our molecular dynamics models are in agreement with reported structures of 3CL^pro^ (15, 17–19, 21) and tightly overlay six X-ray crystallographic structures of SARS-CoV-2 3CL^pro^ in complex with six polyprotein cleavage site sequences (Fig. S3)—raising confidence in our models and interpretations of undescribed specificity determinants for which X-ray crystal structures are not yet reported. On the non-prime side, the P1-Gln side chain is in range to form hydrogen bonds with the 3CL^pro^ domain II residues: Phe140 (1.8 Å), Asn142 (3.5 Å), Ser144 (3.2 Å), His163 (1.8 Å), and Glu166 (2.7 Å) (Figure 2c, e–i, Fig. S5). In these interactions, the amide group of P1-Gln is both a hydrogen bond acceptor and donor through the Oε1 and Nε2, respectively. In addition to P1 side chain interactions, intermolecular hydrogen bonds with 3CL^pro^ protomer B Ser1 are formed by Phe140 (2.1 Å) and Glu166 (1.7 and 3.3 Å) (Fig. 2e). His172 is the third residue that forms an intermolecular hydrogen bond (2.7 Å) with the Ser1 of 3CL^pro^ protomer B. Disruption of these interactions by blocking or modifying the 3CL^pro^ N-terminus has been linked to loss of enzyme activity (21). With the software available, we were unable to perform the simulation for a system having both active sites of protomer A and B simultaneously occupied.

In our previous study (3) and in the current PICS assays, we showed that 3CL^pro^ cleaves after a non-canonical histidine and methionine in P1. This is not without precedence for coronavirus 3C proteases. Cleavage at P1-His by SARS-CoV (−1) 3CL^pro^ was reported by Goetz et al. (22). Chuck et al. (20) further profiled SARS-CoV (−1) 3CL^pro^ substrate specificity at P5 to P3′ positions using 19 × 8 single substitutions and, in addition to P1-Gln and P1-His, the authors identified cleavage at P1-Met. We extended these studies to SARS-CoV-2 3CL^pro^ using massively diverse biologically derived peptide libraries.

**Fig 4:**
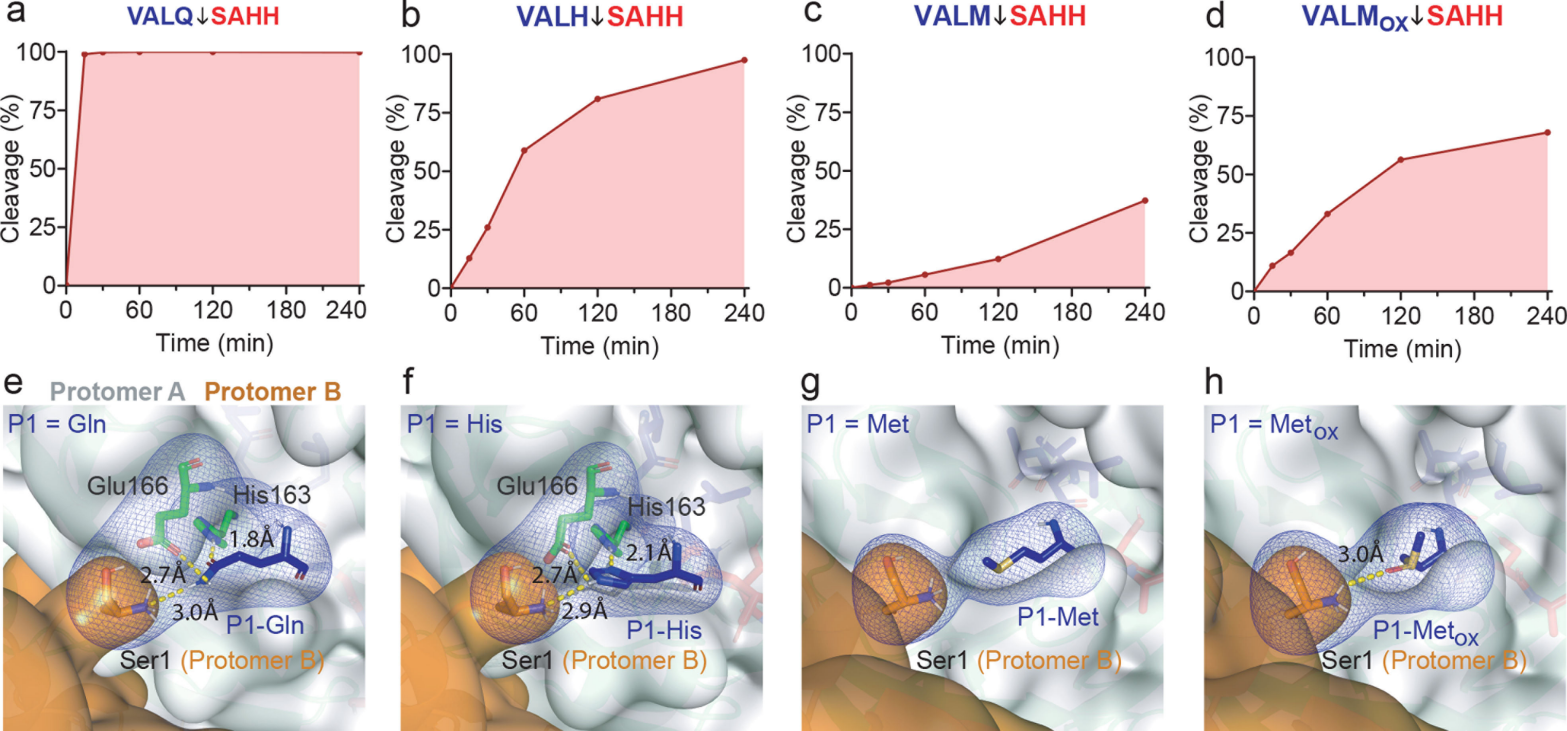
Influence of non-canonical His, Met, and Met_OX_ P1 residues on 3CL^pro^ activity. (a) P1-Gln and (b) the non-canonical P1-His, (c) P1-Met, and (d) P1-Met_OX_ cleavage rates are shown after incubation with 3CL^pro^ (1:50 molar ratio, **E:S**). 3CL^pro^ cleavage sites are indicated by ↓. (e–h) The network of intermolecular H-bond (dashed yellow sticks) interactions between 3CL^pro^ Protomer A Glu166 and His163 (green sticks) amino acid residues (gray surface) and Ser1 in Protomer B (orange surface), with P1-Gln/His/Met/Met_OX_ (blue sticks). H-bond interactions involving the respective P1 residues in the 3CL^pro^ active site predicted by molecular docking simulations are shown.

Essential for cleavage is the Ser1 H-bonding interactions with a P1-Gln (7, 15), involving both the N-terminus of Ser1 and OG that act as a H-bond donor to Glu166 (Fig. 4e). Using synthetic peptides with identical amino acid sequences (AAVAL**X**↓SAHHYAYR) except for P1 (Fig. 4a through d), we found that cleavage at the canonical P1-Gln showed an ^app^(*k*_cat_/*K*_M_) of 1,528 M^−1^s^−1^, followed by P1-His (222 M^−1^s^−1^) and P1-Met (37 M^−1^s^−1^) in the MALDI-TOF assay format. Like the P1-Gln side chain that acts as a hydrogen bond donor and acceptor, P1-His forms a similar hydrogen bond network through its imidazole moiety with His163 (2.1 Å), Glu166 (2.7 Å), and the Ser1 α-amine (2.9 Å) of the protomer B N-terminus (Fig. 4f).

Intriguingly, despite the cleavage of peptides with a P1-Met (ALM↓SAH) and the human native protein substrate MAP4K5 at SKLM↓SENT (3), H-bonding of the Ser1 N-terminus with the P1 side-chain is absent when P1 is Met (Fig. 4g). However, the S1 subsite of 3CL^pro^ accommodates the P1-Met side chain with no unfavorable interactions resulting in a slower cleavage rate than for P1-Gln and P1-His. SARS-CoV-2 induces cell damage by increasing intracellular reactive oxygen species (ROS) levels (23–26). The thiol group of the methionine side chain is one of the main targets of ROS (27, 28) and methionine sulfoxide in native proteins is of significant physiological relevance (29–32). Therefore, we performed chemical oxidation of P1 methionine in the synthetic peptide AAVAL**M**↓SAHHYAYR to compare cleavage rates. The peptide containing the oxidized methionine (Met_OX_) was obtained in >99% yield (Fig. S6), and its cleavage efficiency was 3-fold higher (^app^*k*_cat_/*K*_M_ = 111 M^−1^s^−1^) than for the unmodified peptide (37 M^−1^s^−1^) (Fig. 4c and d). We ascribe the faster cleavage rate of oxidized methionine (P1-Met_OX_) to a 3.0 Å hydrogen bond between the introduced oxygen atom that acts as a H-bond acceptor and the protomer B N-terminal amine group (Fig. 4h). The slight differences in kinetic parameters measured between the different libraries or quenched fluorescent peptides are consistent with the properties of the different assay formats.

### Cleavage site amino acid positional cooperativity

**Fig 5:**
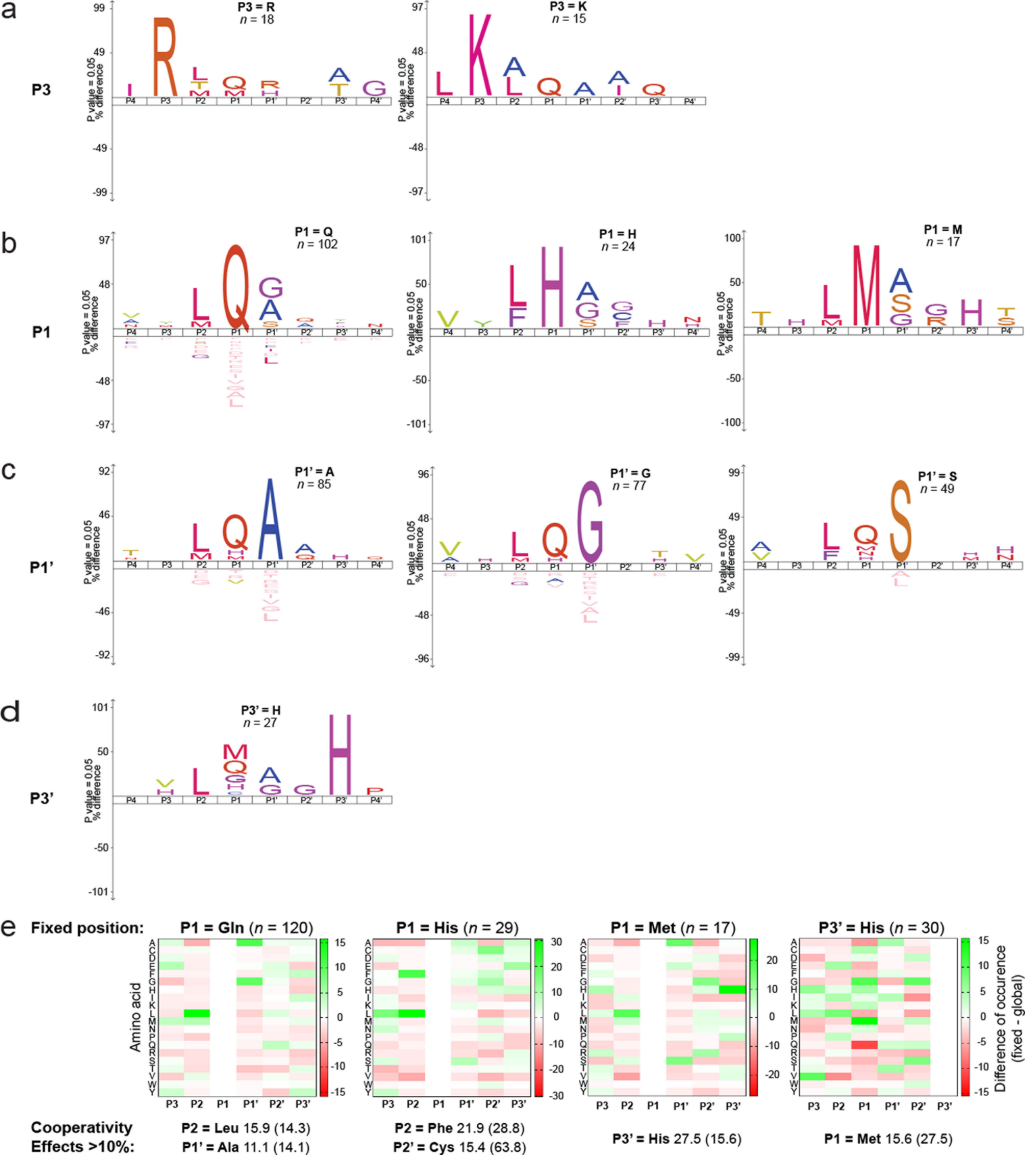
Amino acid cooperativity between peptide positions in 3CL^pro^ cleavage site sequences determined by fixed amino acid and positional analysis. IceLogo plots (a–d) and heat map analyses (e) of cleavage site preferences where the amino acids shown at P3 (a), P1 (b), P1' (c), and P3' (d) were fixed. *n,* number of sites with the fixed amino acid shown. (e) Cooperativity effects between cleavage subsites that surpassed the WebPICS default threshold (10% frequency) are shown with the reciprocal analysis percentage occurrence presented in parentheses.

An advantage of PICS is the large variation of the peptide library. In addition to investigating substrate specificity at individual positions, we sought positional synergism between different residues. To do so, we generated Icelogo motif plots where single amino acids were fixed at selected positions (Fig. 5). Notably, for the noncanonical P1-His, we found that phenylalanine was the preferred amino acid residue at P2 (28.8%) and cysteine was preferred at P2' (15.4%). Indeed, when cysteine was found at P2' then 63.8% of these peptides also had histidine in P1 confirming cooperative stabilizing interactions in the peptide context (Fig. 5b and e). When methionine occupies P1, we found that P3′-His was significantly preferred in 27.5% of peptides (Fig. 5b and e). If histidine was fixed at P3', a reciprocal pair analysis showed that methionine was also its preferred partner with a frequency of 15.6% confirming positive cooperativity. In contrast, for amino acid residues in the canonical cleavage motif (LQ↓G/A/S) the frequency of P1-Gln with P2-Leu and with P1′-Ala/Gly/Ser was only ~11%, just above the 10% baseline, and so does not indicate significant synergism (Fig. 5b).

### Quench fluorescent peptide assay design

**Fig 6:**
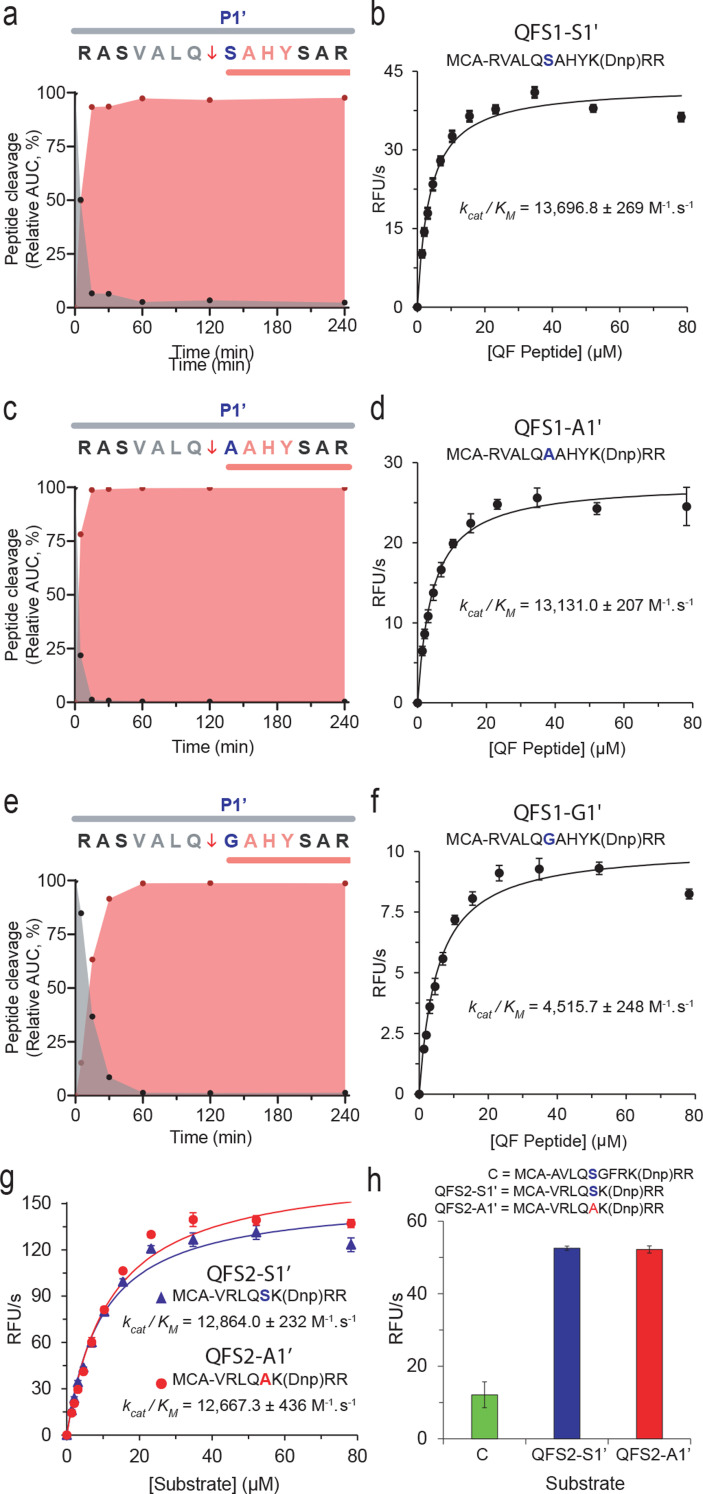
Influence of canonical P1' amino acid residues on the cleavage kinetics of 3CL^pro^ with an optimal P sequence. Cleavage kinetics of VALQ↓XAHY, where the optimal P1' amino acids determined from Table 1 were incorporated at X. (**a, c and **e) Product generation (red) and substrate consumption (gray) after incubation of the peptides with 3CL^pro^ at a ratio of 1:50 (enzyme:substrate) for 5, 15, 30, 60, 120, and 240 min. 3CL^pro^ cleavage sites are indicated by ↓. (**b, d, and **f) Kinetic analysis of the hydrolysis of quenched fluorescent substrate 1 with P1′-Gly1 (QFS1-G1'), P1′-Ser1 (QFS1-S1'), and P1′-Ala1 (QFS1-A1') hydrolysis catalyzed by 0.3 µM 3CL^pro^. (g) Comparison of cleavage rates of 5 μM of optimized QFS2 substrates comparing P1′-Ser with P1′-Ala (QFS2-S1', QFS2-A1') designed for higher solubility and FRET efficiency. (h) Comparison of initial velocities for the hydrolysis of 5 μM of three quenched fluorescent substrates by 3CL^pro^ at 0.3 µM. Control peptide C is the peptide developed for assaying SARS-CoV-1 3CL^pro^ and commonly utilized for SARS-CoV-2 3CL^pro^ cleavage assays. 7-Methoxycoumarin-4-acetyl (MCA), 2,4-dinitrophenyl (Dnp). 3CL^pro^ cleavage sites are indicated by ↓.

Both the commercially available and frequently used, yet insensitive, SARS-CoV-1 3CL^pro^ quenched fluorescent peptide substrate (Mca-AVLQ↓SGFR{Lys(Dnp)}RR-NH_2_) and the improved SARS-CoV-2 3CL^pro^ substrate optimized with non-natural non-prime side amino acids (6) suffer from poor solubility and handling properties. The novel sequence and structural insights provided by PICS and molecular modeling with focussed positional synthetic peptide follow-up enabled the rational design of new SARS-CoV-2 3CL^pro^-specific quenched fluorescent peptide substrates with improved FRET monitoring of 3CL^pro^ activity. We first optimized the prime-side sequence by incorporating a P3′-His and optimizing the P1' residue in VALQ↓**X**AHY by synthesizing P1′-**X** with Ser, Ala, or Gly. P1' Ser and Ala showed the fastest cleavage (Fig. 6a, c and e; Table 1). We next evaluated the respective quenched fluorescent substrate (QFS1) versions of the three peptides: We added the fluorophore 7-methoxycoumarin-4-acetyl (Mca) followed by an arginine residue to the peptide N-terminus and the quencher 2,4-dinitrophenyl (Dnp) to the C-terminus, where it was covalently bonded to a lysine side chain. This was followed by two arginine residues for improved solubility to give Mca-RVALQ↓**X**AHYK(Dnp)RR, where P1′-**X** is Ser (QFS1-S1'), Ala (QFS1-A1') or Gly (QFS1-G1'). 3CL^pro^ showed high specificity for the three new quenched fluorescent substrates with the *k*_cat_*/K*_M_ calculated for QFS1-S1' (13,696.8 M^−1^_·_s^−1^) and QFS1-A1' (13,131.0 M^−1^_·_s^−1^). Notably, these *k*_cat_*/K*_M_ values were three times higher than for QFS1-G1' (4,515.7 M^−1^_·_s^−1^) (Fig. 6b, d and f), confirming the significant enzymatic specificity for Ser and Ala over Gly at P1'.

P3-Arg or Lys were often identified in native human protein substrates by TAILS (3) and here by PICS (Fig. 5a) but only in the GluC library (Fig. 1b), as GluC does not truncate arginine-containing peptides. Therefore, we substituted P3-Ala for Arg for improved solubility. Aiming to increase FRET efficiency further, we shortened the distance between the fluorophore and quencher in version 2 by removing the N-terminal Arg and shortening the prime side from four residues before the quencher to give QFS2-S1' Mca-VRLQ↓SK(Dnp)RR. To precisely measure the preference for P1′-Ser versus P1′-Ala, we also synthesized QFS2-A1' Mca-VRLQ↓AK(Dnp)RR, both of which displayed high *k*_cat_*/K*_M_ values of 12,864.0 M^−1^_·_s^−1^ for QFS2-S1' and 12,667.3 M^−1^_·_s^−1^ for QFS2-A1'. Despite a slightly lower *k*_cat_*/K*_M_ than for QFS1-S1' and QFS1-A1', there was a significant gain in solubility and FRET efficiency, resulting in a strong fluorescence signal of 55 RFUS/s even at low enzyme (0.3 µM) and substrate concentration (5 µM) (Fig. 6h). Our new quenched fluorescent substrates outperform other available 3CL^pro^-specific quenched fluorescent substrates >15-fold in sensitivity without the solubility issues of previous substrates arising from the hydrophobic nature of the donor-acceptor pair.

## DISCUSSION

Using proteome-derived peptide library screens, molecular modeling simulations, and focussed positional peptide libraries, we have detailed the P4–P4' specificity of SARS-CoV-2 3CL^pro^. We show that the P1' amino acids alanine and serine are cleaved 3× faster than glycine, the hydrophobic small amino acids Leu, Ile, or Val prevent any cleavage of otherwise optimal non-prime sequences, and we characterized non-canonical non-prime specificity. We explored the unusual P1-Met specificity discovering enhanced cleavage when in the oxidized state (P1-Met_OX_) and unveiled unexpected amino acid cooperativity at the P1 position, P1-Met with P3′-His and P1-His with P2-Phe, and the importance of the threonine trio in defining this prime side binding in SARS-CoV-2.

To launch these analyses, we constructed three separate peptide libraries from cellular proteomes in the PICS assay (8). Using enzymes with highly specific cut-sites: trypsin (Lys/Arg↓), GluC (Asp/Glu↓), and lysargiNase (↓Lys/Arg) maximized the number of cleavage sites identified (*n* = 816), including the possibility that 3CL^pro^ cuts at the same sites as trypsin, GluC, or lysargiNase. Peptide-docking simulations revealed that stabilizing the substrate prime side occurs exclusively through the 3CL^pro^ catalytic domain I, whereas non-prime side interactions are mediated by catalytic domain II in agreement with crystallographic structures (17, 18, 21). The major 3CL^pro^ prime side stabilizing residues are three consecutive threonine residues Thr24-Thr-25-Thr26 that we term the threonine trio. Using synthetic peptides and MALDI-TOF-MS analyses, we showed that having Leu, Ile, or Val in P1' will completely prevent peptide cleavage by 3CL^pro^, even when the non-prime side has an optimal cleavage sequence. We utilized the statistical power of the 816 sites identified to design optimized quenched fluorescent peptides superior to conventional peptide assays in solubility and sensitivity for the high-sensitivity detection of 3CL^pro^ activity.

Trypsin libraries exclude the detection of lysine and arginine at P1 and nearby positions since semi-tryptic peptides ending or starting close to a 3CL^pro^ site are not detectable. Indeed, the P3 arginine specificity we identified was only possible using the library prepared with GluC. The trypsin library revealed the exclusion of P1' Leu, Ile, Glu, and Asp (*n* = 415). The GluC library showed that Lys and Arg were also excluded at P1' along with Leu, Val, and Pro (*n* = 328). These findings are consistent with the present models and prior X-ray crystallographic structures that revealed the 3CL^pro^ subsite S1' does not accommodate bulky residues due to steric hindrance imposed by Thr25, Leu27, and His41 side chains. P1'–P4' residues extend over the threonine trio, maximizing main-chain interactions. Additionally, the side chain of Thr25 stabilizes the P3' side chain through hydrogen bond interactions, favoring H-bond donor/acceptor residues at P3', such as His. Notably, MERS 3CL^pro^ lacks the threonine trio (Fig. 2b).

For P1-Gln side chain stabilization, X-ray structures show the critical interaction with Glu166 supported by Ser144 and His163 (17, 19). The Ser144 hydroxyl group forms a hydrogen bond with the carbonyl oxygen of the P1-Gln side chain. Simultaneously, a second hydrogen bond interaction can be established between the main chain nitrogen of Ser144 and the backbone oxygen atom of P1. In addition to Ser144 Oγ, His163 Nε2 donates a hydrogen bond to the P1-Gln side chain oxygen. These interactions make the side chain nitrogen atom of P1-Gln a better hydrogen bond donor since the electron donation from Nε2 to the adjacent carbonyl group (*n* → π*) is increased (*see* Fig. S4). Consequently, a strong hydrogen bond between Glu166 Oε1 and P1-Gln Nε2 is favored. Glu166 is known to play a key role in the catalytic activity of 3CL^pro^ (32). We hypothesize that the Ser144 and His163 residues favor the Glu166/P1-Gln interaction. In addition, the hydrogen bond network correctly orientates the P1 main chain carbonyl for subsequent nucleophilic attack by the Ser144 Sγ to promote hydrolysis of the peptide bond.

Like glutamine, the non-canonical P1-His is the only other residue that can form the same hydrogen bonding network in which its pyrrole-like nitrogen assumes the role of hydrogen bond donor and the pyridine-like nitrogen forms a hydrogen bond as an acceptor. Although asparagine contains a polar amide group in its side chain, P1-Asp stabilization and simultaneous nucleophilic attack by Ser144 Sγ would not occur due to the short length of the Asp side chain. P1-Met is cleaved at a slower rate due to the lack of stabilizing hydrogen bond interactions involving its side chain. However, when oxidized, the side chain of P1-Met can interact with the N-terminal amino group of 3CL^pro^ protomer B, promoting more efficient cleavage. We are unaware of other examples of oxidized methionine favoring cleavage. It is well known that ROS targets the thiol group of methionines (27). Recent studies have shown that ROS production is markedly elevated in COVID-19 patients and is related to disease pathogenesis and progression (23–26). This rise in intracellular ROS could increase the range of 3CL^pro^ substrates in the cell and improve the cleavage of proteins containing a P1-Met to form kinetically accessible substrates.

Developing next-generation antiviral therapies, vaccines, and viral protease assays for inhibitor development is fundamental to characterizing the molecular mechanisms of pathogenesis and virulence and, ultimately, in combating COVID-19. Currently, the most widely employed quenched fluorescent substrate has a *k*_cat_/*K*_M_ = 859 M^−1^s^−1^ and was optimized using non-natural amino acids (Ac-Abu-Tle-Leu-Gln-ACC) (6) to be a marked improvement over the SARS-CoV-1 3CL^pro^ peptide substrate early in the pandemic. The structural insights gleaned from global analysis of >800 cleavage sites identified by PICS and in-depth molecular simulation modeling allowed us to design two new quenched fluorescent substrates Mca-VRLQSK(Dnp)RR and Mca-VRLQAK(Dnp)RR. Notably, these peptide substrates are designed from natural amino acids, facilitating their synthesis and application. By displaying >15× improved sensitivity with a *k*_cat_/*K*_M_ of 13,696.8 M^−1^_·_s^−1^ and superior solubility and handling properties, the Mca-VRLQSK(Dnp)RR quenched fluorescent substrate can be applied in various assay formats, including those for high- throughput drug screening.

## Data Availability

Further information and requests for resources and reagents can be directed to Professor Chris Overall (chris.overall@ubc.ca).

## SUPPLEMENTAL MATERIAL

Supplemental material jvi.00049-24-s0001.pdf Table S1, Figures S1 to S6, and computer code.

ASM does not own the copyrights to Supplemental Material that may be linked to, or accessed through, an article. The authors have granted ASM a non-exclusive, world-wide license to publish the Supplemental Material files. Please contact the corresponding author directly for reuse.
