# Supplementary material for: Optimization of quenched fluorescent peptide substrates of SARS-CoV-2 3CLpro main protease (Mpro) from proteomic identification of P6—P6' active site specificity
Source: J Virol. 2024 May 14;98(6):e00049-24. doi: 10.1128/jvi.00049-24 (PMC11237654; doi:10.1128/jvi.00049-24)
Supplement: Supplemental material — Table S1, Figures S1 to S6, and computer code. [file jvi.00049-24-s0001.pdf]

TABLE S1. Cα RMSD (Å) between the docked PICS peptide VALQ↓GAHY and the crystal structure of six native substrates from SARS-CoV-2 replicase polyproteins in complex with 3CL^{pro} mutant (H41A).

Cleavage substrate	PDB ID	RMSD (Å)
nsp4|5	7DVP	0.29
nsp5|6	7DVW	0.37
nsp6|7	7DVX	0.30
nsp9|10	7DVY	0.25
nsp14|15	7DW0	0.16
nsp15|16	7DW6	0.23

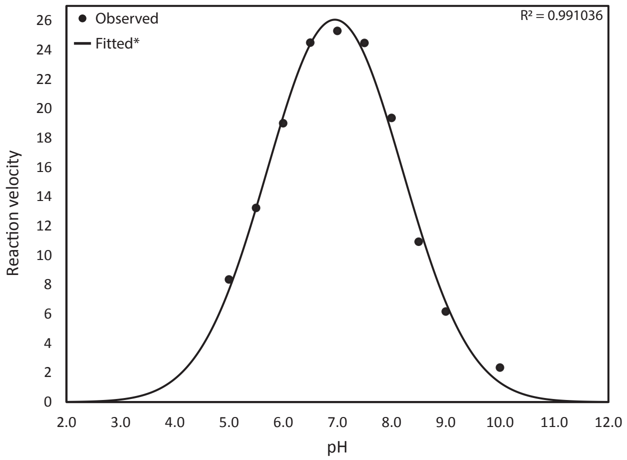

Figure S1. Effect of pH on 3CLpro activity. The peptide hydrolysis reaction was monitored by time course measurement of fluorescence increase upon hydrolysis of the quenched fluorescent substrate, MCA-VRLQAK(Dnp)RR, (λex = 320 nm, λem = 405 nm) at each pH, ranging from 5.0 to 10.0.

* The curve was fitted using the equation below, where "a" is the amplitude of the curve, "b" is the position of the center of the curve, and "c" is associated with the width.

$$y = a \times e^{-0.5\times\left(\frac{x-b}{c}\right)^2}$$

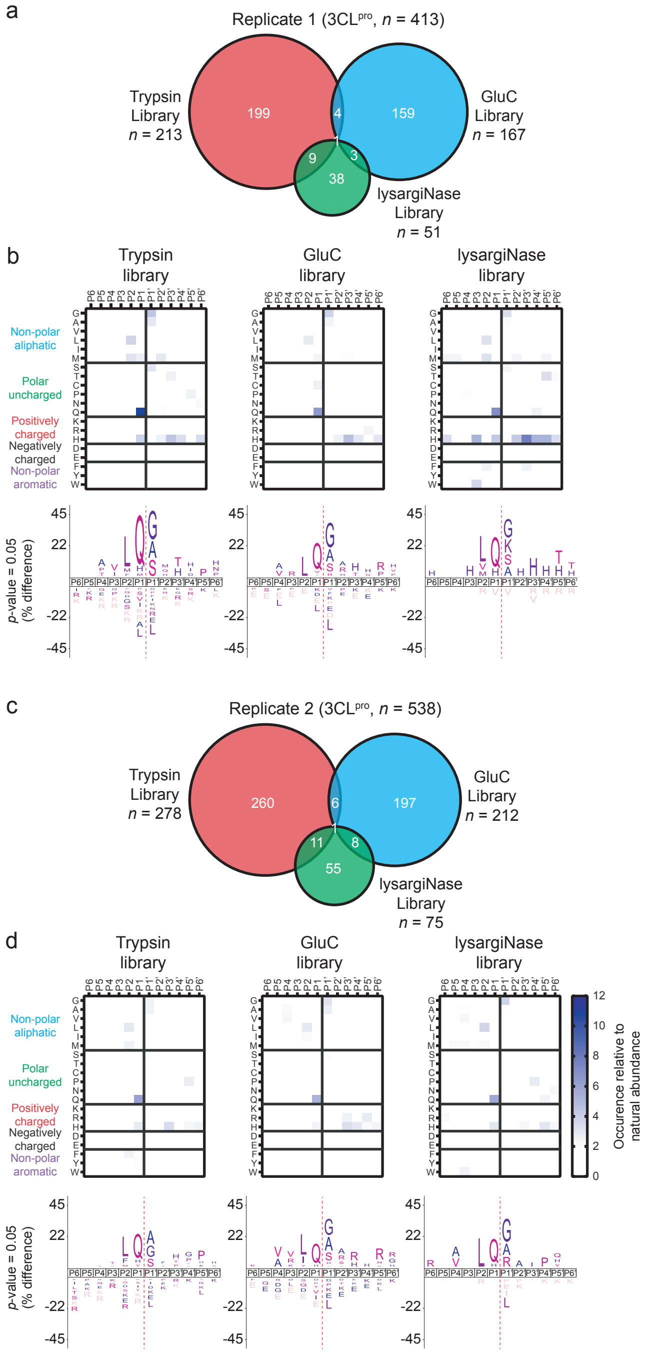

Figure S2. The number of unique 3CL^{pro} cleavage sites identified in 2 biological replicates from 3 PICS libraries. Venn diagrams displaying overlap of identified 3CL^{pro} cleavage sites and heatmaps showing enrichment (> 2-fold natural abundance) of residues at P6 – P6' positions for each PICS library.

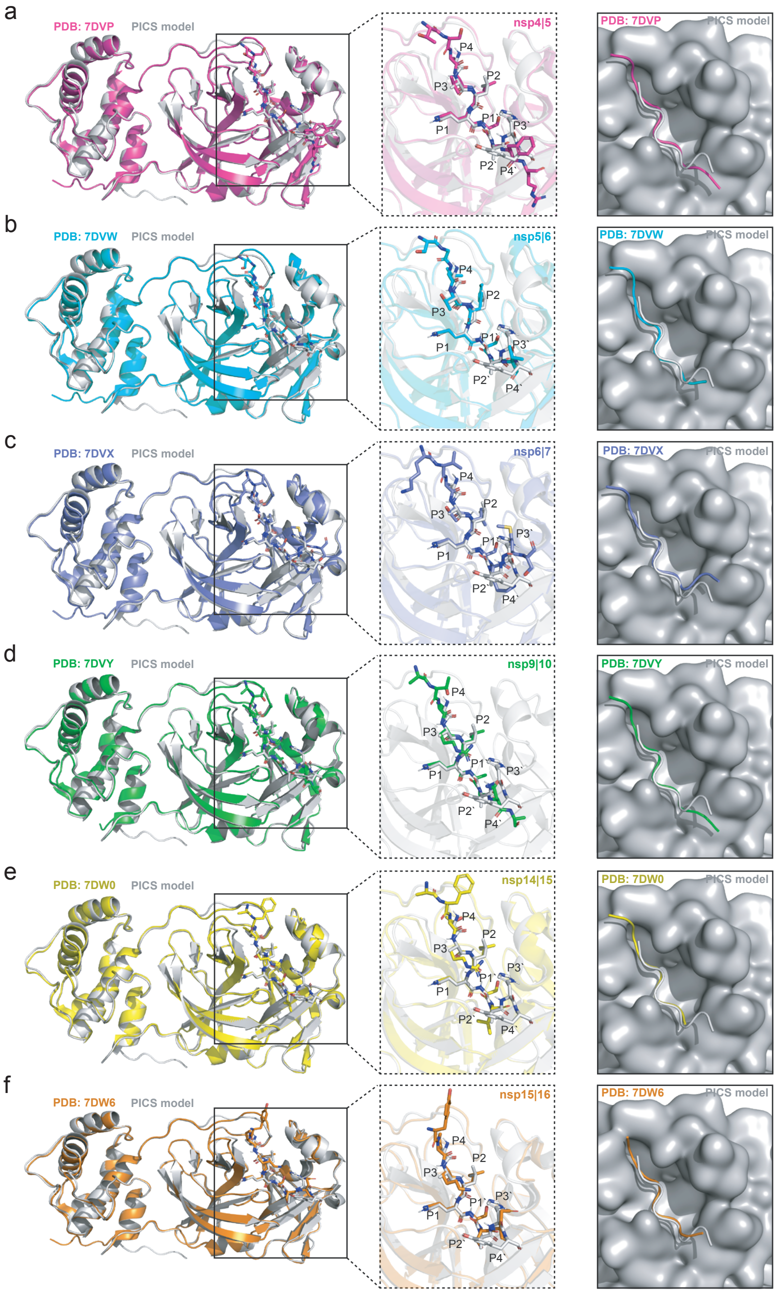

Figure S3. Structural model overlays of the peptide identified by PICS, VALQ↓GAHY, and x-ray crystallography structures of six native polyprotein substrates from SARS-CoV-2. 3CL^{pro} (H41A) and the polyprotein P4 – P4' of the cut site spanning the boundary between the non structural proteins (nsps) shown in color are overlaid with the PICS peptide VALQ↓GAHY docked with the modelled 3CL^{pro} structure (grey) shown in **Figure 2.**

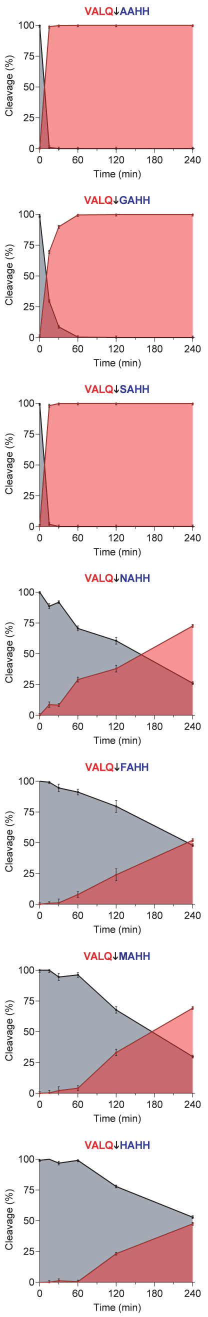

Figure S4. MALDI-TOF peptide cleavage assay. Product generation (red) and substrate consumption (black) after incubation of the peptides as shown with 3CL^{pro} at a ratio of 1:50 (enzyme:substrate) for 5, 15, 30, 60, 120 and 240 min. 3CL^{pro} cleavage sites are indicated by ↓.

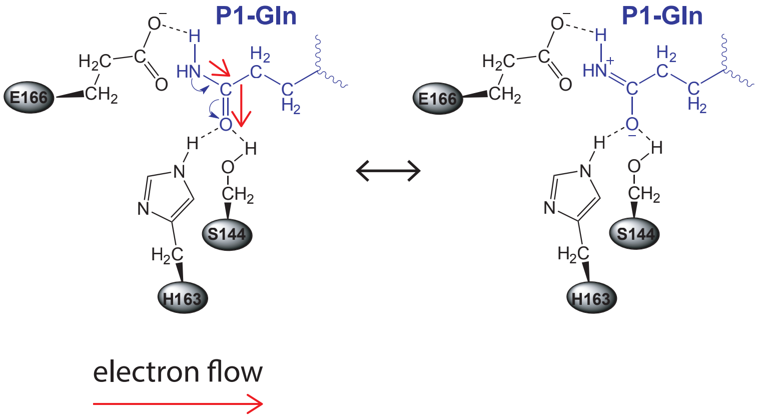

Figure S5. H-bond network between P1 sidechain and Glu166, His163 and Ser144.

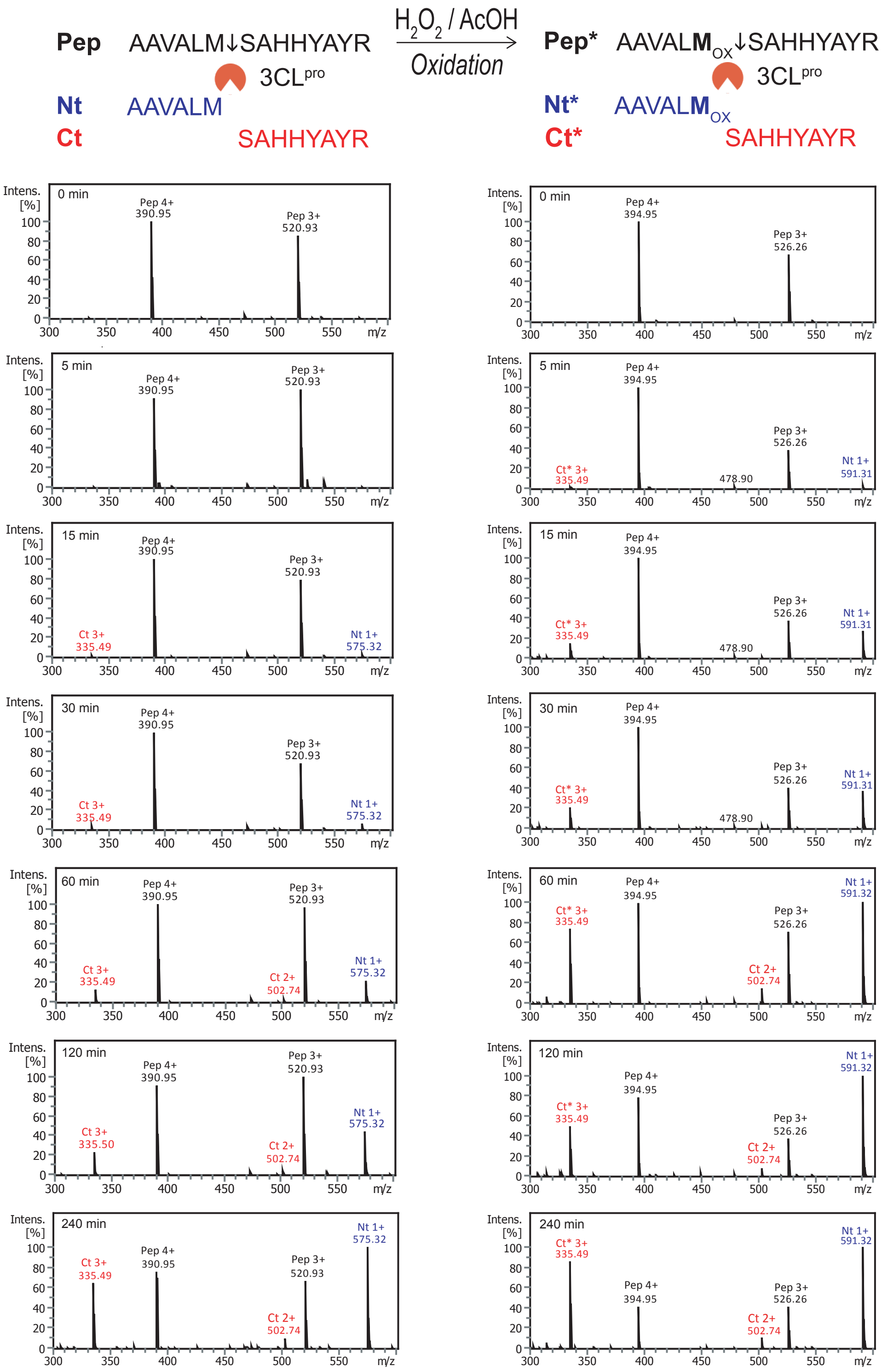

Figure S6. Cleavage analysis of peptides at a noncanonical P1 methionine by 3CL^{pro}. [a] MALDI-TOF-MS spectra of intact (left) and oxidized (right) synthetic peptide AAVALM↓SAHHYAYR incubated with 3CL^{pro} for the indicated times. $\mathbf{M}_{OX}$, oxidised methionine.

Rosetta Prepacking Structure and Flags.

(a) Prepacking structure.

Command line: $ROSETTA_BIN/FlexPepDocking.mpi.linuxgccrelease @prepack_flags

Where prepack_flags =

```
-s $PICS_input/3CL_PICS_pep.pdb
-ex1
-ex2aro
-ex2
-ex1aro
-database $ROSETTA_DB_Path
-scorefile prepack.score.sc
-flexpep_score_only
-nstruct 1
-out:path:pdb $PICS_output
-out:path:score $PICS_output
-use_truncated_termini
```

(b) High-resolution peptide-protein docking.

Command line: $ROSETTA_BIN/FlexPepDocking.mpi.linuxgccrelease @abinitio_flags

Where abinitio_flags =

```
-s $PICS_input/3CL_PICS_pep_ppk.pdb   # prepacked structure as input
-lowres_abinitio                      # Low-resolution ab-initio folding and docking
-pep_refine                           # Refine low-resolution models produced using -
lowre_abinitio flag
-flexpep_score_only                   # Read in a complex, score it and output interface
statistics
-ex1                                  # Add extra side-chain rotamers
-ex2aro                               # Add extra side-chain rotamers
-ex1aro
-ex2
-use_truncated_termini                # Will not add extra OXT/Hs at termini if not in
input structure
-frag3 $PICS_input/frags/frags.3mers.offset   # 3mer / 5mer / 9mer fragments
files for ab-initio peptide docking.
-flexPepDocking:frag5 $PICS_input/frags/frags.5mers.offset
#frag9 input/frags/ptbp1_offset.200.9mer
-flexPepDocking:frag5_weight 0.25
#flexPepDocking:frag9_weight 0.1
-constraints:cst_weight 2
-constraints:cst_fa_file $PICS_input/constraints
-constraints:cst_file $PICS_input/constraints
-constraints:cst_fa_weight 2
-score:weights ref2015_cst
-out:pdb
```

```
-out:path:pdb $PICS_output
-out:file:scorefile $PICS_output/PICS_complex.sc
-nstruct 50000                    # Number of models to create in the simulation
-flexPepDocking:receptor_chain A
-flexPepDocking:peptide_chain D
```

(c) Constraint file.

AtomPair SG 145A CA 310D FLAT_HARMONIC 2.0 0.25 2.0

(d) Flat harmonic constraint penalty function.

$$\boldsymbol{f}(dist) = \begin{cases} \mathbf{0}, \text{if } distance\ between\ Ser145\ S\gamma\ and\ P1\ C\alpha\ \leq 4 \\ \left(\frac{(\boldsymbol{dist\ between}\ \mathbf{Ser145\ S\gamma\ and\ P1\ C\alpha}) - \mathbf{4.0}}{\mathbf{0.25}}\right)^{\mathbf{2}}, \text{otherwise} \end{cases} .$$
